# Commentary: Parental Depressive Symptoms as a Predictor of Outcome in the Treatment of Child Internalizing and Externalizing Problems

**DOI:** 10.3389/fpsyt.2019.00533

**Published:** 2019-07-26

**Authors:** Talha Tahir, Alameen Damer, Michael Wong

**Affiliations:** ^1^Bachelor of Health Sciences Program, McMaster University, Hamilton, ON, Canada; ^2^Psychology Department, University of Wisconsin–La Crosse, La Crosse, WI, United States

**Keywords:** depression, behavior, children, internalizing, externalizing

## Introduction

Parental depression is a risk factor for the development of child internalizing (e.g., depression, anxiety) and externalizing problems (e.g., aggression, delinquency) (1–4). Eckshtain et al. (5) sought to explore whether parental depression negatively influences the outcome of interventions aimed at treating clinically significant internalizing and externalizing problems. The authors investigated 142 children and analyzed weekly child and parent reported trajectories of change in behavior as a result of the interventions. They found that, in children with internalizing problems, those with less depressed parents showed symptom declines, whereas children with more depressed parents showed an increase. In children with externalizing problems, Eckshtain et al. (5) found steeper symptom declines in those with more depressed patients. However, parental depression was not measured prospectively, and other confounding factors potentially affecting children’s response to the interventions were not considered. This makes it difficult to ascertain whether the observed results are attributable solely to parental depression.

## Possible Changes in Parental Depression

Eckshtain et al. (5) found that, in children of depressed parents, internalizing problems increased over the course of treatment. In theory, the interventions employed by the researchers should have decreased internalizing symptoms, or at the very least, held them at baseline. Therefore, the observed increase in internalizing symptoms is an unexpected finding. One possibility for this finding is a change in parental depression. Eckshtain et al. (5) did not measure parental depression post-intervention; therefore, an increase in parental depression over the course of treatment is plausible and may have caused a corresponding increase in child internalizing symptoms. Therefore, the apparent impotence of the intervention may result from its effects on children’s internalizing problems being overpowered by worsening parental depression. Parental depression has been found to be associated with internalizing problems in children by numerous studies. Weissman et al. (4) found that children of depressed parents are three times more likely to develop anxiety disorder and major depression. Other studies have corroborated these results and have shown a significant increase in the risk for child psychopathology in children of depressed parents (1–3). Additionally, a mean treatment duration of 232.19 days in the internalizing symptoms group makes it plausible that parental depression may have deviated from baseline (5). The severity of presenting symptoms in both major depressive disorder and dysthymic disorder has been reported to vary over time, with periods of depressive symptoms punctuated by normal mood (6, 7). To prevent the potential effects of variable parental depressive symptoms from affecting their results, Eckshtain et al. (5) could have prospectively measured parental depression in a fashion similar to child internalizing symptoms.

## Other Potential Influencing Factors

Other factors that may have influenced the relationship observed between parental depression and child internalizing and externalizing problems are discussed below. We comment on a few factors that, if taken into consideration by Eckshtain et al. (5), may have strengthened the observed relationships.

### In Children

Sibling relationships have been implicated as potential influencing factors in children’s internalizing and externalizing problems. Buist and Vermande (8) found that children with conflictual sibling relationships reported significantly more internalizing and externalizing problems than children with harmonious relationships. Other studies have described sibling conflict to be predictive of increased childhood anxiety and depression and associated with maternal reports of delinquency and maternal and teacher reports of aggression (9, 10). Evidence also suggests that peer victimization may influence child internalizing and externalizing behavior. Literature suggests an association between peer victimization and behavior problems such as social problems, aggression, and externalizing problems (11, 12). Assessing children’s perception of their sibling relationships and peer victimization may have helped the authors ensure that these factors were balanced and invariant throughout treatment. An increase in any of the aforementioned factors during the treatment period could have caused a corresponding elevation of internalizing and externalizing problems in children, thereby potentially impacting the results observed by Eckshtain et al. (5).

### In Parents

Parental psychopathology has also been found to influence child behavior problems. Eckshtain et al. (5) measured parental psychopathology with the Depression Symptoms Dimension of the Brief Symptom Inventory. While the depression symptoms dimension is adequate for measuring baseline parental depression, it ignores the comorbidity of depression with other psychopathologies (e.g., anxiety disorder, substance abuse disorder, and personality disorders (13–16). Children of parents with these comorbid psychopathologies have been reported to experience more behavior problems; for example, children of substance abusers have been shown to score higher on internalizing and externalizing scales than demographically matched non-referred children (17). Therefore, it is possible that these psychological conditions were distributed asymmetrically across parents with elevated depressive symptoms compared to parents without depressive symptoms, producing effects that may have been misattributed to baseline parental depression levels. Other potential factors that were not taken into consideration but have been suggested to play a role in child internalizing and externalizing problems include marital conflict and domestic violence. Moylan et al. (18) found that children exposed to domestic violence had increased levels of internalizing and externalizing problems in adolescence. They also found that exposure to child abuse in conjunction with domestic violence was an even stronger predictor of higher externalizing and internalizing problems.

## Conclusion

Eckshtain et al. (5) did not prospectively measure parental depression during or after the intervention, nor did they control for potentially influencing factors such as sibling relationships, peer victimization, and marital conflict on internalizing and externalizing problems. Without knowing the degree to which the aforementioned factors are present within the sample, it is difficult to ascertain their effects on the results. Children with exposure to these risk factors may be more resistant to the interventions prescribed by Eckshtain et al. (5). Therefore, for future studies of this nature, it is recommended that researchers account for as many potentially influencing factors as possible.

## Author Contributions

All authors listed have made a substantial, direct, and intellectual contribution to the work, and approved it for publication.

## Conflict of Interest Statement

The authors declare that this manuscript was written in the absence of any commercial or financial relationships that could be construed as a potential conflict of interest.

## References

[1] Brennan PA Hammen C Katz AR Le Brocque RM Maternal depression, paternal psychopathology, and adolescent diagnostic outcomes. J Consult Clin Psychol (2002) 70:1075–85. 10.1037/0022-006X.70.5.1075 12362958

[2] Lewinsohn PM Olino TM Klein DN Psychosocial impairment in offspring of depressed parents. Psychol Med (2005) 35:1493–503. 10.1017/S0033291705005350 PMC1351338 16164773

[3] Lieb R Isensee B Höfler M Pfister H Wittchen H-U Parental major depression and the risk of depression and other mental disorders in offspring: a prospective-longitudinal community study. JAMA Psychiatry (2002) 59:365–74. 10.1001/archpsyc.59.4.365 11926937

[4] Weissman MM Wickramaratne P Nomura Y Warner V Pilowsky D Verdeli H Offspring of depressed parents: 20 years later. Am. J Psychiatry (2006) 163:1001–8. 10.1176/ajp.2006.163.6.1001 16741200

[5] Eckshtain D Marchette LK Schleider J Evans S Weisz JR Parental depressive symptoms as a predictor of outcome in the treatment of child internalizing and externalizing problems. J Abnorm Child Psychol (2019) 47:459–74. 10.1007/s10802-018-0446-2 PMC6261702 29808395

[6] Otte C Gold SM Penninx BW Pariante CM Etkin A Fava M Major depressive disorder. Nat Rev Dis Prim (2016) 2:16065. 10.1038/nrdp.2016.65 27629598

[7] Sansone RA Sansone LA Dysthymic disorder: forlorn and overlooked? Psychiatry (Edgmont) (2009) 6:46–51. PMC2719439 19724735

[8] Buist KL Vermande M Sibling relationship patterns and their associations with child competence and problem behavior. J Fam Psychol (2014) 28:529–37. 10.1037/a0036990 24866727

[9] Stocker CM Burwell RA Briggs ML Sibling conflict in middle childhood predicts children’s adjustment in early adolescence. J Fam Psychol (2002) 16:50–7. 10.1037/0893-3200.16.1.50 11915410

[10] Garcia MM Shaw DS Winslow EB Yaggi KE Destructive sibling conflict and the development of conduct problems in young boys. Dev Psychol (2000) 36:44–53. 10.1037/0012-1649.36.1.44 10645743

[11] Reijntjes A Kamphuis JH Prinzie P Boelen PA Van Der Schoot M Telch MJ Prospective linkages between peer victimization and externalizing problems in children: a meta-analysis. Aggress Behav (2011) 37:215–22. 10.1002/ab.20374 21433031

[12] Kim YS Leventhal BL Koh Y-J Hubbard A Boyce WT School bullying and youth violence: causes or consequences of psychopathologic behavior? JAMA Psychiatry (2006) 63:1035–41. 10.1001/archpsyc.63.9.1035 16953006

[13] Brady KT Killeen TK Brewerton T Lucerini S Comorbidity of psychiatric disorders and posttraumatic stress disorder. J Clin Psychiatry (2000) 61:22–32. 10795606

[14] Bleich A Koslowsky M Dolev A Lerer B Post-traumatic stress disorder and depression: an analysis of comorbidity. Br J Psychiatry (1997) 170:479–82. 10.1192/bjp.170.5.479 9307701

[15] Overbeek T Schruers K Vermetten E Griez E Comorbidity of obsessive–compulsive disorder and depression: prevalence, symptom severity, and treatment effect. J Clin Psychiatry (2002) 63:1106–12. 10.4088/JCP.v63n1204 12523869

[16] Corruble E Ginestet D Guelfi JD Comorbidity of personality disorders and unipolar major depression: a review. J Affect Disord (1996) 37:157–70. 10.1016/0165-0327(95)00091-7 8731079

[17] Stanger C Higgins ST Bickel WK Elk R Grabowski J Schmitz JOY Behavioral and emotional problems among children of cocaine- and opiate- dependent parents. J Am Acad Child Adolesc Psychiatry (1999) 38:421–8. 10.1097/00004583-199904000-00015 10199114

[18] Moylan CA Herrenkohl TI Sousa C Tajima EA Herrenkohl RC Russo MJ The effects of child abuse and exposure to domestic violence on adolescent internalizing and externalizing behavior problems. J Fam Violence (2010) 25:53–63. 10.1007/s10896-009-9269-9 20495613 PMC2872483

